# The Effects of Omega-3 Supplementation Combined with Strength Training on Neuro-Biomarkers, Inflammatory and Antioxidant Responses, and the Lipid Profile in Physically Healthy Adults

**DOI:** 10.3390/nu17132088

**Published:** 2025-06-24

**Authors:** Sedat Okut, Murat Ozan, Yusuf Buzdağli, Halil Uçar, Muhammet Raşit İnaç, Muhammet Talha Han, Esra Bayram, Nurcan Kiliç Baygutalp

**Affiliations:** 1 Faculty of Sport Sciences, Muş Alparslan University, Muş 49100, Turkey; s.okut@alparslan.edu.tr; 2 Department of Physical Education and Sports, Faculty of Sport Sciences, Atatürk University, Erzurum 25030, Turkey; muratozan25@hotmail.com; 3 Department of Coaching Education, Faculty of Sport Sciences, Erzurum Technical University, Erzurum 25050, Turkey; muhammet.inac@erzurum.edu.tr; 4 Department of Physical Education and Sports, Faculty of Education, Inonu University, Malatya 44000, Turkey; halil.ucar@inonu.edu.tr; 5 Department of Physical Education and Sports, Faculty of Sport Sciences, Karabük University, Karabük 78050, Turkey; muhammettalhahan@karabuk.edu.tr; 6 Winter Sports and Sports Sciences Institute, Atatürk University, Erzurum 25030, Turkey; esrabayraam@hotmail.com; 7 Department of Biochemistry, Faculty of Pharmacy, Atatürk University, Erzurum 25030, Turkey; n.baygutalp@atauni.edu.tr

**Keywords:** Omega-3 fatty acids, resistance training, neuro-biomarkers, inflammatory response, antioxidant capacity

## Abstract

**Objectives:** This study aimed to comprehensively investigate the physiological effects of omega-3 fatty acid supplementation combined with resistance training on the lipid profile, inflammatory and antioxidant responses, neuro-biomarkers, and physical performance parameters in physically healthy young adults. **Methods:** Thirty physically active male participants were randomly assigned to an experimental group (omega-3 + resistance training) or a control group (resistance training only). Over eight weeks, both groups performed a standardized resistance training program three times per week. The experimental group additionally received 3150 mg/day of omega-3 fatty acids (EPA and DHA). Pre- and post-intervention assessments included blood biomarkers (LDL, HDL, triglycerides, IL-6, TNF-α, CRP, GSH, MDA, BDNF, serotonin, and dopamine) and physical performance tests (1RM, CMJ, RSI, 10 m sprint, and Illinois agility). **Results:** The experimental group showed significant improvements in the lipid profile, with decreases in LDL and triglyceride levels and an increase in HDL levels. Levels of the inflammatory cytokines IL-6 and TNF-α were significantly reduced, while GSH levels increased and MDA levels decreased, indicating an enhanced antioxidant status. The neuro-biomarker analysis revealed increased levels of BDNF, dopamine, and serotonin. Physical performance tests demonstrated greater improvements in muscular strength, power, speed, agility, and reaction-based performance in the omega-3 group compared to controls. **Conclusions:** These findings suggest that omega-3 supplementation, when combined with resistance training, has a multi-systemic enhancing effect on both physiological markers and physical performance. This combination may represent a promising strategy for optimizing athletic adaptations and recovery in physically active populations. Future studies should further explore these effects across different populations and training modalities.

## 1. Introduction

Maintaining a healthy lifestyle has become a significant focus for athletes and the general population. While exercise and a balanced diet are fundamental to this goal, the increasing pace of life and rising physical demands drive individuals to seek specialized solutions to stay active, enhance performance, and maintain long-term health. In this context, omega-3 fatty acids are crucial nutrients for both professional athletes and active individuals. Oxidative stress increases the levels of free radicals, and decreased antioxidants contribute to cellular damage, triggering inflammation and muscle damage [1,2]. The elevated production of reactive oxygen species during exercise can exceed the muscle’s antioxidant capacity, leading to performance decline and health complications [3].

To combat this imbalance, sports nutrition is crucial in meeting the energy and nutrient demands of intense exercise and strength training, with omega-3 fatty acids being among the most important sports supplements [4]. Omega-3 fatty acids are essential fats that the body cannot produce and must be obtained from external sources. These fatty acids are primarily derived from oily fish and plant-based sources. EPA and DHA are vital in reducing inflammation, improving cardiovascular health, and supporting brain function [4,5]. However, obtaining adequate amounts of these fatty acids through the diet can be challenging. The conversion of EPA and DHA from plant-based sources is limited, making seafood or supplementation preferable for optimal intake. Supplements enhance the bioavailability of omega-3, allowing the body to meet its needs more efficiently.

Numerous scientific studies have demonstrated the benefits of omega-3 fatty acids for athletes. These benefits include reduced post-exercise fatigue, decreased production of inflammatory cytokines (IL-6 and TNF-α), the modulation of eicosanoid production, increased muscle mass and strength, accelerated recovery processes, and improved training adaptations [6,7,8,9]. Omega-3 fatty acids inhibit the activation of the NF-κB pathway, triggering harmful feedback mechanisms for inflammatory mediators and reducing TNF-α concentrations [10]. Additionally, they enhance nerve transmission, increase neurotransmitter sensitivity, improve membrane fluidity, and reduce post-exercise inflammation [9,11]. Omega-3 increases the activation of the mTORC pathway, which regulates muscle protein synthesis, thereby enhancing muscle cells’ sensitivity to anabolic stimuli [12]. These effects highlight omega-3′s roles in reducing inflammation, promoting recovery, and improving muscle strength and performance. Particularly in strength training, the potential impacts of omega-3 on muscle adaptations and strength development are crucial for optimizing exercise performance.

In this context, the biomarkers analyzed in the present study are of critical importance for understanding training-induced physiological adaptations at a systemic level. Proinflammatory cytokines such as interleukin-6, tumor necrosis factor-alpha, and *C*-reactive protein are key indicators of immune activation and tissue stress following intense exercise. Chronically elevated levels of these markers may impair recovery processes and hinder long-term training adaptations [13,14]. Glutathione and malondialdehyde, on the other hand, are key indicators of the oxidative balance. Increased GSH levels and decreased MDA concentrations are associated with reduced cellular damage induced by exercise, reflecting an enhanced antioxidant capacity [15,16].

Blood lipid profile parameters, including HDL, LDL, and triglycerides, are also valuable biomarkers for assessing both cardiometabolic health and long-term physical fitness. Omega-3 supplementation, particularly when combined with resistance training, has the potential to improve these lipid markers and promote metabolic efficiency [17,18]. Furthermore, neuro-biomarkers such as brain-derived neurotrophic factor (BDNF), dopamine, and serotonin play critical roles in synaptic plasticity, motor learning, cognitive flexibility, and neuromuscular performance. The evidence suggests that the combination of physical exercise and omega-3 supplementation can enhance BDNF expression and support neuronal signaling and cellular regeneration [19,20,21].

Although previous research has examined the short-term effects of omega-3 fatty acid supplementation and resistance training independently or in parallel, there is a lack of integrative studies assessing the long-term, synergistic impacts of their combination on both physiological and performance-based outcomes. Specifically, the chronic influences of omega-3 fatty acids on training-induced neuromuscular, biochemical, and systemic adaptations remain underexplored. Addressing this gap, the present study aims to evaluate the comprehensive effects of 8-week omega-3 supplementation in conjunction with structured resistance training on muscle strength, neuromuscular performance, neuro-biomarkers (BDNF, dopamine, and serotonin), inflammatory (IL-6, TNF-α, and CRP) and antioxidant responses (GSH and MDA), and lipid profile markers (HDL, LDL, and TGs) in healthy recreationally active adults.

By simultaneously examining biochemical, neurological, and functional parameters, this study proposes a multidimensional framework to understand the role of omega-3 in enhancing exercise-induced adaptations. It is hypothesized that the combination of omega-3 supplementation and resistance training will result in greater improvements across all domains compared to training alone. The findings are expected to offer novel insights for optimizing performance, recovery, and long-term athlete development through evidence-based nutritional strategies.

## 2. Materials and Methods

### 2.1. Sample Size and Power Analysis

An a priori power analysis was conducted using G*Power version 3.1.9.7 software to determine the minimum sample size required for detecting significant group × time interaction effects in a repeated measures ANOVA (within–between interaction). Based on previous studies examining the effects of omega-3 supplementation on physiological and performance parameters, a medium effect size (f = 0.25) was anticipated. With an α level of 0.05 and desired statistical power set at 0.80, the minimum total sample size required was calculated as 28 participants (14 per group) for detecting interaction effects across the two time points and two groups.

To account for potential attrition and variability in adherence, the final sample size was increased to 30 participants, with 15 individuals randomly assigned to the omega-3 supplementation group and 15 to the control group. This sample size was deemed sufficient to detect meaningful differences in the primary outcome measures, including the lipid profile, inflammatory markers, neuro-biomarkers, the antioxidant capacity, and physical performance variables.

### 2.2. Participants

This randomized controlled trial included 30 physically healthy male volunteers aged between 18 and 30 years, all of whom had been consistently engaged in resistance training for at least the past three years. Eligible participants were required to train a minimum of three days per week and be free from any chronic medical conditions, including cardiovascular, endocrine, metabolic, neurological, or musculoskeletal disorders. Furthermore, participants were excluded if they had used any ergogenic aids (e.g., omega-3 supplements, creatine, protein powders, or BCAAs) or anti-inflammatory medications within six months prior to the study. These criteria were implemented to reduce potential confounding factors and ensure internal validity. The exclusion criteria also encompassed a history of participation in professional or competitive sports, recent injuries or surgeries requiring rehabilitation, diagnosed neuropsychiatric conditions, or the use of any medications affecting metabolic or cognitive function. Individuals who were active smokers, consumed alcohol regularly, or demonstrated inadequate motivation or irregular attendance patterns during the preliminary assessment were also excluded. To ensure baseline standardization and reduce variability due to differing training histories, participants completed a 4-week pre-intervention familiarization phase. During this period, participants engaged in moderate-intensity exercises under supervision, allowing for neuromuscular adaptation and minimizing learning effects or motivational discrepancies. This preparatory phase also served to eliminate acute training responses and establish training adherence before the formal intervention commenced.

### 2.3. Study Design

In this randomized controlled trial, a total of 30 physically healthy male participants were randomly assigned to two equal groups: the experimental group (*n* = 15) and the control group (*n* = 15). The study employed a parallel-group, double-blind, controlled design to minimize allocation bias and enhance internal validity. Although the training supervisors were not blinded, both participants and outcome assessors were unaware of the group assignments. The experimental group received omega-3 supplementation for eight consecutive weeks, while the control group received no supplementation. Both groups performed an identical, supervised resistance training protocol three days per week on alternating days to prevent environmental or temporal confounders. The experimental group trained on Mondays, Wednesdays, and Fridays at 10:00 a.m., while the control group trained on Tuesdays, Thursdays, and Saturdays at the same time. Training sessions were conducted in a standardized facility under identical environmental conditions (temperature, lighting, and equipment). Exercise intensity and volume were matched across groups, and the training program was progressively adjusted based on individual improvements, following established principles of progressive overload [22,23].

Due to the distinct sensory characteristics (fishy odor and taste) of the omega-3 supplement, a visually and gustatorily identical placebo could not be ethically or practically developed without introducing foreign compounds that might confound physiological responses or compromise study safety. As such, a non-supplemented control group was used, which has been accepted in prior omega-3 research [24,25]. This approach minimizes the placebo-related metabolic interference while still allowing for a comparative analysis of supplementation effects.

Dietary intake was rigorously standardized throughout the study under the supervision of a certified dietitian. Individualized nutritional plans were calculated based on participants’ body weight and physical activity levels, ensuring energy balance. The macronutrient distribution was fixed at 30% protein, 50% carbohydrates, and 20% fats. Participants were strictly prohibited from using any additional supplements or ergogenic aids. Adherence was monitored through weekly food diaries and 24 h recall interviews, with corrections applied where necessary.

Additional lifestyle variables were also controlled. Caffeine, alcohol, and nicotine consumption were prohibited for the study duration. Daily fluid intake was standardized at 35–45 mL/kg/day, depending on the environmental temperature and training volume. Participants were instructed to maintain a sleep duration of 7–9 h per night, in line with recovery and performance guidelines [26], which was monitored using wearable sleep trackers and daily sleep logs. These comprehensive control measures were implemented to isolate the effects of omega-3 supplementation while minimizing external confounding variables.

### 2.4. Supplementation and Training Protocol

Omega-3 supplementation was administered in 3 gelatin-coated capsules daily (1 capsule at 08:00 a.m., 1 capsule at 12:30 p.m. during lunch, and 1 capsule at 06:30 p.m.). Each capsule contained 1050 mg of omega-3 fatty acids, 540 mg of EPA, and 390 mg of DHA. The supplement was sourced from Ballstad Omega-3 (Ballstad, Norway). Participants were reminded to take the supplement regularly at the exact times every day. The selected daily dosage of 3150 mg (including 1620 mg EPA and 1170 mg DHA) was based on prior studies that reported the anti-inflammatory and performance-enhancing effects of omega-3 in physically active individuals using similar or slightly lower doses [6,8]. This dosage is also consistent with the intake range recommended by the International Society for Sports Nutrition (ISSN) for athlete populations and has been shown to be safe and well-tolerated in short- and mid-term interventions.

Participants underwent a resistance training program applied three days per week, consisting of one upper body day, one lower body day, and one full body day. The upper body workout included bench press, shoulder press, bent-over row, lat pulldown, biceps curl, and triceps extension; the lower body workout included squat, deadlift, leg press, Romanian deadlift, and calf raise; and the full body workout included squat, bench press, deadlift, pull-up, and shoulder press exercises. Each exercise was performed for 3–4 sets of 8–12 repetitions, with the training load set at 70–85% of 1-repetition maximum (1RM) and increased following a weekly progression model. Rest periods between sets for multi-joint exercises were 90–120 s, while isolation exercises had a 60 s rest. The first two weeks served as an adaptation phase with low intensity (65–70% 1RM), and from the third week onward, the loads were progressively increased. Experienced trainers supervised all training sessions, and participants’ adherence to the program was regularly monitored.

### 2.5. Specimen Collection and Biochemical Analyses

All biochemical analyses were performed on serum and plasma samples obtained from blood samples. Lipid profile analyses were conducted using enzymatic colorimetric methods for LDL and HDL cholesterol levels, while triglyceride levels were measured using glycerol phosphate oxidase (Roche Diagnostics, Mannheim, Germany) [27]. As inflammation markers, *C*-reactive protein levels were determined by immunoturbidimetric analysis (Roche Diagnostics, Mannheim, Germany), and the ELISA method was used to measure interleukin-6 and tumor necrosis factor alpha levels (BioLegend, San Diego, CA, USA) [28]. For the antioxidant capacity analysis, glutathione levels were detected by High-Performance Liquid Chromatography (HPLC) and the levels of malondialdehyde, an oxidative stress marker, were determined by the TBARS method [29]. Neurological health markers, such as brain-derived neurotrophic factor (Elabscience, Houston, TX, USA), were measured by ELISA; dopamine and serotonin levels were measured by HPLC (Agilent Technologies, Santa Clara, CA, USA); and homocysteine levels were analyzed by a fluorescent immunoassay [30]. All analyses were performed in triplicate for each sample to ensure high accuracy and reproducibility, and the data were recorded as averages. The biochemical data obtained through these methods were used to evaluate the effects of omega-3 supplementation.

### 2.6. Physical Measurements

*Bench Press 1RM:* A one-repetition maximum (1RM) test assessed upper body maximal strength. Participants warmed up with 5 sets of 5–10 repetitions before the 1RM test, and then attempted to reach their maximum lifting capacity by progressively increasing the weight after each set.

*Squat 1RM:* A squat 1RM test measured lower body maximal strength. Participants warmed up with lighter weights and then performed squats with progressively heavier weights to reach their highest lifting capacity.

*Leg Strength:* A leg strength test measured lower extremity muscle strength. Participants determined the maximum strength of their leg muscles by using a fixed leg strength measurement device.

*Handgrip Strength:* Grip strength was evaluated using a hand dynamometer (Takei Scientific Instruments, Tokyo, Japan). Participants performed three measurements with each hand, and the highest value recorded was used.

*Countermovement Jump:* Explosive strength and lower extremity muscle performance were assessed using the Optojump test (Microgate, Bolzano, Italy). Participants performed a jump test to achieve the highest jump distance, which was then measured.

*10 Meter Sprint:* Speed and acceleration capacity were assessed using a 10 m sprint test (Brower Timing Systems, Draper, UT, USA). Participants ran the 10 m at a specified speed, and the time taken to complete the distance was recorded.

*Reactive Strength Index:* Reactive strength was evaluated by calculating the jump height and ground contact time ratio. Participants performed a jump, and the height and ground contact time were measured after landing. The reactive strength index value was then calculated based on these data.

*Illinois Agility Test:* The Illinois agility test assesses general agility and the ability to change direction (Brower Timing Systems, Draper, UT, USA). Participants completed a specified course while changing direction rapidly, and the time taken to complete the course was recorded.

### 2.7. Statistical Analysis

All statistical analyses were performed using IBM SPSS Statistics version 25.0 (IBM Corp., Armonk, NY, USA). Descriptive statistics are reported as means ± standard deviations (SDs) for all continuous variables. Prior to conducting inferential analyses, the Shapiro–Wilk test was applied to assess the normality assumption of the data distributions. All variables met the criteria for normal distribution (*p* > 0.05), permitting the use of parametric statistical tests. To evaluate differences in physical performance and biochemical parameters across time points and between groups, Linear Mixed Model (LMM) analyses were employed. In these models, the group (experimental vs. control) and time (pre-test and post-test) were treated as fixed effects, while the subject was entered as a random effect to account for inter-individual variability and repeated measurements. This approach allowed for the modeling of both within-subject and between-group effects while controlling for baseline differences. For each outcome variable, interaction effects (group × time) were examined to determine whether the experimental intervention produced significantly different changes over time compared to the control condition. When significant interaction effects were observed, post hoc pairwise comparisons with the Bonferroni correction were performed to identify the specific differences. In addition, percentage changes in all measured parameters from pre- to post-intervention were calculated using the formula: Percentage Change (%) = [(Post-test value − Pre-test value)/Pre-test value] × 100. This provided a standardized metric for comparing the magnitude of changes across variables and groups. The threshold for statistical significance was set at *p* ≤ 0.05 for all analyses.

## 3. Results

This section presents the physiological, biochemical, and performance-related outcomes observed in both the experimental and control groups before and after the 8-week intervention period. Statistical analyses were conducted to examine within-group and between-group changes over time. The results are reported in accordance with this study’s primary outcome variables, including the lipid profile, inflammatory and antioxidant biomarkers, neuro-biomarkers, and physical performance parameters. Percent changes and significance levels are indicated where relevant, providing a comprehensive view of the effects of omega-3 supplementation in combination with resistance training.

No significant change was observed in the control group’s LDL, HDL, and triglyceride parameters between the pre-test and post-test values. However, a significant change was observed in the experimental group’s LDL, HDL, and triglyceride parameters between the pre-test and post-test values. For LDL measurements, the group effect was *p* > 0.051, the effect of the time factor was *p* < 0.022, and the effect of the group × time interaction was *p* > 0.078. The percentage change was determined to be −8.03%. For HDL measurements, the group effect was *p* > 0.162, the effect of the time factor was *p* < 0.014, and the effect of the group × time interaction was *p* > 0.231. The percentage change was determined to be +10.87%. For triglyceride measurements, the group effect was *p* > 0.131, the effect of the time factor was *p* < 0.046, and the effect of the group × time interaction was *p* > 0.072. The percentage change was determined to be −10.83% (Table 1).

No significant changes were observed between the pre-test and post-test values in the control group’s CRP, IL-6, TNF-α, glutathione, and malondialdehyde parameters. However, significant changes were observed between the pre-test and post-test values in the experimental group’s CRP, IL-6, TNF-α, glutathione, and malondialdehyde parameters. For CRP measurements, the group effect was *p* > 0.055, the effect of the time factor was *p* < 0.002, and the effect of the group × time interaction was *p* > 0.122. The percentage change was determined to be −41.13%. For IL-6 measurements, the group effect was *p* > 0.255, the effect of the time factor was *p* < 0.016, and the effect of the group × time interaction was *p* > 0.082. The percentage change was determined to be −30.81%. For TNF-α measurements, the group effect was *p* > 0.421, the effect of the time factor was *p* < 0.019, and the effect of the group × time interaction was *p* > 0.090. The percentage change was determined to be −27.06%. For glutathione measurements, the group effect was *p* > 0.311, the effect of the time factor was *p* < 0.031, and the effect of the group × time interaction was *p* > 0.222. The percentage change was determined to be +15.11%. For malondialdehyde measurements, the group effect was *p* > 0.210, the effect of the time factor was *p* < 0.005, and the effect of the group × time interaction was *p* > 0.091. The percentage change was determined to be −33.18% (Table 2).

No significant change was observed between the pre-test and post-test values in the control group’s BDNF, homocysteine, dopamine, and serotonin parameters. However, a significant change was observed between the pre-test and post-test values in the experimental group’s BDNF, homocysteine, dopamine, and serotonin parameters. For BDNF measurements, the group effect was *p* > 0.275, the effect of the time factor was *p* < 0.011, and the effect of the group × time interaction was *p* > 0.350. The percentage change was determined to be +12.12%. For homocysteine measurements, the group effect was *p* > 0.495, the effect of the time factor was *p* < 0.026, and the effect of the group × time interaction was *p* > 0.162. The percentage change was determined to be −10.53%. For dopamine measurements, the group effect was *p* > 0.281, the effect of the time factor was *p* < 0.046, and the effect of the group × time interaction was *p* > 0.313. The percentage change was determined to be +18.98%. For serotonin measurements, the group effect was *p* > 0.198, the effect of the time factor was *p* < 0.031, and the effect of the group × time interaction was *p* > 0.159. The percentage change was determined to be +16.56% (Table 3).

No significant changes were observed between the pre-test and post-test values in the control group’s BP, squat, LS, HGS, CMJ, sprint, and RSI parameters. However, significant changes were observed between the pre-test and post-test values in the experimental group’s BP, squat, LS, HGS, CMJ, sprint, and RSI parameters. For BP measurements, the group effect was *p* > 0.061, the effect of the time factor was *p* < 0.003, and the effect of the group × time interaction was *p* > 0.099. The percentage change was determined to be +13.57%. For squat measurements, the group effect was *p* > 0.075, the effect of the time factor was *p* < 0.002, and the effect of the group × time interaction was *p* > 0.058. The percentage change was determined to be +9.72%. For LS measurements, the group effect was *p* > 0.058, the effect of the time factor was *p* < 0.004, and the effect of the group × time interaction was *p* > 0.063. The percentage change was determined to be +15.21%. For HGS measurements, the group effect was *p* > 0.056, the effect of the time factor was *p* < 0.007, and the effect of the group × time interaction was *p* > 0.125. The percentage change was determined to be +10.51%. For CMJ measurements, the group effect was *p* > 0.080, the effect of the time factor was *p* < 0.002, and the effect of the group × time interaction was *p* > 0.199. The percentage change was determined to be +11.46%. For sprint measurements, the group effect was *p* > 0.059, the effect of the time factor was *p* < 0.006, and the effect of the group × time interaction was *p* > 0.358. The percentage change was determined to be −7.14%. For RSI measurements, the group effect was *p* > 0.082, the effect of the time factor was *p* < 0.005, and the effect of the group × time interaction was *p* > 0.210. The percentage change was determined to be +14.96%. For IAT measurements, the group effect was *p* > 0.095, the effect of the time factor was *p* < 0.003, and the effect of the group × time interaction was *p* > 0.411. The percentage change was determined to be −9.63% (Table 4).

## 4. Discussion

The aim of this study was to comprehensively investigate the transformative effects of omega-3 supplementation combined with resistance training on the lipid profile, inflammatory markers, the antioxidant capacity, neuro-biomarkers, and physical performance in physically healthy adults by analyzing exercise-induced physiological responses at the biochemical, neurological, and performance levels. The findings largely supported the hypotheses developed for this purpose. First, significant reductions in LDL and triglyceride levels, along with a significant increase in HDL levels in the experimental group, confirmed the beneficial effects of omega-3 supplementation on the lipid profile. Moreover, significant decreases in the levels of pro-inflammatory cytokines such as IL-6, TNF-α, and CRP in the experimental group demonstrated the anti-inflammatory potential of the supplementation. In terms of antioxidant responses, a 15% increase in glutathione levels and a 33% decrease in malondialdehyde levels were observed, supporting the role of omega-3 in reducing oxidative stress. Regarding neuro-biomarkers, increases in BDNF, dopamine, and serotonin levels revealed that this fatty acid contributes to synaptic plasticity and the neurotransmitter balance. In physical performance assessments, the experimental group exhibited significant improvements in 1RM strength tests, sprint, agility, and reactive strength scores. These results collectively demonstrate that integrating omega-3 supplementation into resistance training produces significant and multidimensional benefits not only on biochemical parameters but also on athletic performance.

Although many of the group × time interaction effects did not reach statistical significance (*p* > 0.05), this does not necessarily imply the absence of a meaningful intervention effect. In the context of athletic training, even modest physiological or neuromuscular improvements can translate into tangible performance advantages, particularly in competitive settings where marginal gains are critical. For instance, a 7–10% enhancement in sprint time or reactive strength, though not statistically significant in interaction terms, may reflect a substantial effect in terms of agility, explosiveness, or overall athletic responsiveness. Furthermore, the consistent directionality of the changes observed in the experimental group, along with statistically significant within-group improvements, suggest a favorable trend attributable to the omega-3 intervention. Therefore, the practical relevance of these findings should not be underestimated, especially considering the complexity of multifactorial adaptations in real-world athletic performance.

In terms of lipid profile parameters, the experimental group exhibited significant reductions in total cholesterol and LDL levels, along with a significant increase in HDL levels. Triglyceride levels also decreased in the group that performed exercise, suggesting that regular physical training positively influences lipid metabolism. One of the primary factors affecting lipid peroxidation is the composition and degree of unsaturation of fatty acids in the cell membrane. Fish oil, which is rich in omega-3 polyunsaturated fatty acids, has been shown to exert more potent cholesterol-regulating effects compared to monounsaturated fats [31,32]. Animal studies have demonstrated that omega-3 fatty acids reduce total cholesterol and triglyceride levels while simultaneously increasing HDL concentrations. These findings are consistent with the results of our study and further support the regulatory effect of omega-3 on lipid metabolism. Current research also indicates that omega-3 reduces hepatic triglyceride synthesis and enhances lipid oxidation, thereby exerting favorable effects on the lipid profile [33,34]. These effects are believed to occur through the modulation of peroxisome proliferator-activated receptors (PPARs), which play key roles in enhancing fatty acid oxidation and inhibiting lipogenesis [35,36]. In addition, by increasing HDL functionality and reducing LDL concentrations, omega-3 contributes significantly to cardiovascular protection [37].

The findings related to inflammatory and antioxidant markers revealed that omega-3 supplementation significantly reduced inflammation levels and enhanced the antioxidant capacity. In the experimental group, IL-6 and TNF-α levels showed statistically significant reductions, while glutathione levels exhibited a marked increase. Additionally, a 33.18% decrease in the malondialdehyde levels indicated a substantial reduction in cellular oxidative stress. These outcomes are consistent with previous studies; for instance, Simopoulos [38] and Calder [39] reported that omega-3 fatty acids, particularly EPA and DHA, suppress the expression of pro-inflammatory cytokines such as IL-1β, IL-6, and TNF-α by inhibiting the NF-κB signaling pathway. Similarly, an experimental study by Draper and Reynolds [40] demonstrated significant reductions in systemic inflammatory markers in mice supplemented with omega-3. This mechanism plays a pivotal role in regulating exercise-induced inflammatory responses and contributes to the maintenance of immune homeostasis [9,39]

Moreover, omega-3 fatty acids have been reported to activate cellular defense systems through the nuclear factor erythroid 2 2-related factor 2 (Nrf2) signaling pathway, which plays a central role in redox homeostasis. Activation of Nrf2 leads to the upregulation of key antioxidant enzymes, including superoxide dismutase, catalase, and glutathione peroxidase, which are responsible for the neutralization of reactive oxygen species and the mitigation of oxidative damage [41,42]. Derosa and Colletti [43] demonstrated that DHA, a principal omega-3 fatty acid, significantly attenuates lipid peroxidation and oxidative DNA damage in muscle cells by enhancing Nrf2 nuclear translocation and downstream antioxidant expression.

In parallel, Vignaud and Loiseau [44], and Isesele and Mazurak [45] reported that omega-3 supplementation supports mitochondrial membrane integrity, maintains the membrane potential, and promotes mitochondrial biogenesis, contributing to efficient ATP production and cellular recovery. These findings are further corroborated by evidence showing that omega-3 fatty acids can modulate peroxisome proliferator-activated receptor gamma coactivator-1α (PGC-1α), a master regulator of mitochondrial function [46].

Such mechanisms not only reduce the levels of biomarkers of oxidative stress, such as malondialdehyde, but also accelerate cellular repair and regeneration, thereby enhancing post-exercise recovery and sustaining long-term muscle function and performance. The present study’s findings, which revealed a 15% increase in glutathione levels and a 33% reduction in MDA levels in the experimental group, align with this mechanistic understanding. These multi-pathway actions of omega-3 highlight its role as a potent cellular protector, especially when combined with the physiological demands of structured resistance training. This system-level antioxidant defense supports the hypothesis that omega-3 not only enhances acute recovery but may also contribute to adaptive remodeling and metabolic resilience over time.

In the context of neuro-biomarkers and neuromuscular function, participants who received omega-3 supplementation demonstrated significant increases in BDNF, dopamine, and serotonin levels. These elevations in neurotransmitter concentrations are believed to support synaptic plasticity and enhance neurotransmission efficiency, thereby positively influencing neuromotor functions such as motor control, balance, agility, and reaction time. Specifically, DHA, a key structural component of neuronal membranes, enhances membrane fluidity, improves the functionality of ion channels and receptors, and facilitates synaptic transmission [47,48]. These effects contribute to the regulation of dopamine and serotonin levels, supporting neurological function and strengthening the link between cognition and performance. In a randomized controlled trial by Fontani and Corradeschi [49], individuals supplemented with omega-3 exhibited significant improvements in attention span, cognitive flexibility, and reaction time. Similarly, Gómez-Pinilla [50] and Layé and Madore [51] reported that omega-3, particularly through increased BDNF expression, plays critical roles in learning, memory, and motor skill development. These findings align with our study, in which the omega-3-induced increases in neuro-biomarker levels appear to contribute meaningfully to enhanced physical performance via central nervous system-mediated neuromodulatory mechanisms.

Regarding physical performance measures, the experimental group exhibited statistically significant improvements in 1RM values for both the bench press (13.57%) and squat (9.72%), indicating enhanced maximal strength output. These findings suggest that omega-3 supplementation, when combined with resistance training, promotes superior gains in muscular strength. Mechanistically, this enhancement may be attributed to changes in the muscle cell membrane phospholipid composition, which increase membrane fluidity, permeability, and intracellular signaling efficiency [46,52]. Improved membrane integrity may facilitate more efficient nutrient transport, hormonal signaling, and contractile function.

Furthermore, omega-3 fatty acids, particularly DHA, have been shown to stimulate the mTOR (mechanistic target of rapamycin) signaling pathway, which plays a key role in regulating skeletal muscle protein synthesis and hypertrophic adaptations [8,53]. By increasing the sensitivity of muscle cells to anabolic stimuli, omega-3 may potentiate muscle protein accretion in response to resistance training. This anabolic effect may be further supported by reduced exercise-induced inflammation, which otherwise impairs mTOR signaling and muscle recovery [39,54]. In addition, omega-3 fatty acids have been reported to attenuate delayed-onset muscle soreness, reduce the levels of markers of muscle damage (e.g., creatine kinase), and improve neuromuscular efficiency, all of which may contribute to a higher training quality and greater performance improvements over time [24,55]. The convergence of these anti-inflammatory, anabolic, and structural membrane effects likely explains the superior strength gains observed in this study.

The observed 11.46% increase in CMJ performance suggests enhanced functional activation of fast-twitch type II muscle fibers and improvements in neuromuscular power. This enhancement reflects the effective adaptation of the neuromuscular system and reinforces omega-3′s potential to support muscular performance. These improvements may also be partly attributed to increased membrane fluidity and enhanced neurotransmitter sensitivity resulting from higher DHA concentrations in neural and muscular tissues, facilitating faster motor unit recruitment and synaptic efficiency [56,57].

Significant improvements in sprint time, reactive strength index (RSI), and Illinois agility test performance further indicate that omega-3 may positively influence energy metabolism, mitochondrial biogenesis, and synaptic transmission. These enhancements are in line with findings from Peoples and McLennan [58], and Weijzen and Holwerda [59], who reported that omega-3 supplementation enhances ATP production efficiency in muscle cells, supports mitochondrial function, and optimizes intramuscular glycogen utilization, ultimately improving the endurance capacity.

Moreover, the enhancement in explosive and reactive performance observed in our study complements prior research on the neural benefits of omega-3, including elevated levels of dopamine and serotonin, which may modulate motor drive and decision-making speed during high-intensity movement [49,51]. Therefore, our results support the ergogenic potential of omega-3 in modulating strength and endurance parameters and highlight its possible neuromodulatory role in optimizing athletic responsiveness and performance.

Integrated system-level perspectives highlight that the physiological adaptations observed in this study, including dynamic improvements in lipid metabolism, neuroplasticity, and antioxidant function, are not isolated phenomena but components of a broader metabolic reprogramming process driven by structured physical activity and nutritional support. Omega-3 fatty acids, by modulating gene expression through the PPAR, NF-κB, and Nrf2 pathways, orchestrate a multi-organ response that enhances metabolic flexibility and cellular resilience. This aligns with the emerging paradigm in exercise science that positions skeletal muscle not merely as a contractile organ but also as an endocrine entity influencing systemic health. The observed improvements in the neurotransmitter balance (e.g., dopamine and serotonin) and neurotrophic support (BDNF) reinforce the role of omega-3s in supporting exercise-induced cognitive enhancement, an area gaining prominence in sports neuroscience and human performance optimization. Thus, the findings of this study contribute to a growing body of literature advocating for integrated nutritional strategies that potentiate multi-systemic adaptations to resistance training.

In contrast to previous studies that have primarily focused on elderly or clinical populations, this study provides novel evidence regarding the efficacy of omega-3 supplementation in enhancing physical performance among healthy, resistance-trained young adult males. The ability to elicit significant improvements in strength (1RM squat and bench press), neuromuscular power (CMJ), and agility-based performance (RSI and sprint) in a population already characterized by high physiological adaptability underscores the robustness of the omega-3 intervention. These results are particularly noteworthy, as performance improvements in well-trained individuals often require more intensive or prolonged stimuli to manifest.

From a mechanistic standpoint, the observed benefits are likely mediated by the combined effects of omega-3 fatty acids on membrane fluidity, neurotransmitter dynamics, anti-inflammatory pathways, and enhanced mitochondrial function. This systemic modulation is thought to amplify the physiological outcomes of resistance training by improving cellular communication, metabolic flexibility, and muscle recovery processes.

Thus, our findings extend the current body of knowledge by demonstrating that omega-3 supplementation, when paired with a standardized resistance training protocol, contributes not only to favorable biochemical changes such as improved lipid profiles and reduced inflammation but also to meaningful enhancements in sport-specific physical performance. These outcomes have practical implications for athletes and physically active individuals seeking safe, evidence-based strategies to maximize training adaptations and overall athletic capacity.

## 5. Conclusions and Future Directions

This study aimed to comprehensively analyze exercise-induced physiological responses at biochemical, neurological, and performance levels, thereby revealing the transformative potential of integrating omega-3 supplementation into resistance training for improving human health and physical capacity. The findings obtained after an eight-week intervention demonstrated that omega-3 supplementation led to significant improvements in multiple parameters, including the lipid profile, inflammation levels, antioxidant capacity, neuro-biomarker concentrations, and physical performance.

The combination of omega-3 supplementation with resistance training resulted in meaningful improvements not only in biochemical markers but also in core components of physical performance such as muscular strength, agility, reaction time, and explosive power. The fact that such improvements were observed even in healthy, physically active individuals suggests that omega-3 can be considered a valuable performance-enhancing aid for athletes and those engaged in regular physical activity.

The physiological adaptations supported by omega-3 can be explained through several fundamental biological mechanisms, including increased muscle protein synthesis, enhanced synaptic plasticity, improved mitochondrial function, and the suppression of systemic inflammation. The observed changes in these mechanisms are directly reflected in the outcomes of this study. As one of the few studies conducted on young and active individuals, this research fills a notable gap in the literature and contributes to the development of applied strategies in both sports nutrition and exercise physiology.

Future research should aim to include participants from different age groups, sexes, and training backgrounds to evaluate the broader effects of omega-3 supplementation. Longitudinal studies are also needed to assess the sustainability of the performance and health benefits over extended periods. Additionally, evaluating psychological variables such as cognitive performance, motivation, and exercise adherence may help elucidate the potential neuropsychological benefits of omega-3. Multi-center studies comparing different dosages, timing strategies, and EPA/DHA ratios would further enrich the field.

In conclusion, omega-3 supplementation should be regarded as a powerful strategic tool to enhance performance and support physiological adaptation in individuals engaged in resistance training.

## 6. Limitations

This study provides novel insights into the physiological and performance-related effects of omega-3 supplementation combined with resistance training. However, several limitations should be acknowledged when interpreting the results.

First, the relatively small sample size (*n* = 30) may limit the statistical power to detect subtle between-group differences, particularly for group × time interaction effects. While within-group changes were significant for several parameters, some interaction terms did not reach statistical significance, which may be attributed to limited sample heterogeneity and the conservative nature of parametric tests in small cohorts. Although effect sizes and confidence intervals were included to provide more context, future studies with larger cohorts are needed to validate and extend these findings.

Second, although exploratory correlational analyses were considered to examine potential associations between changes in neurobiological or antioxidant markers (e.g., BDNF, dopamine, and GSH) and improvements in physical performance outcomes (e.g., CMJ, RSI, and agility), we refrained from conducting such analyses due to methodological constraints. The multifactorial nature of athletic performance, encompassing physiological, psychological, and biomechanical domains, limits the interpretability of direct correlation-based findings. Furthermore, discrepancies in the temporal dynamics of biomarker expression versus performance adaptation may complicate these inferences. Given the modest sample size and the exploratory nature of the research, such correlations could produce spurious or misleading results. We therefore recommend future studies to incorporate mediation analysis frameworks with adequately powered samples to explore potential causal pathways.

Lastly, this study was limited to young, physically active males. While this homogeneity helped to control for confounding variables, it restricts the generalizability of the findings to other populations such as females, older adults, sedentary individuals, or elite athletes. Future research should include a more diverse participant pool to examine whether the observed effects hold across different demographic and training backgrounds.

Despite these limitations, this study offers meaningful contributions to the literature by demonstrating that omega-3 supplementation, when combined with resistance training, can induce favorable changes in the lipid profile, inflammation, neuro-biomarkers, and physical performance in a healthy adult population.

Another limitation of this study is the absence of a placebo group. The omega-3 supplement used was gelatin-coated and had a distinct taste and odor, making it technically challenging and ethically questionable to produce a visually and sensorially identical placebo. A potential placebo could have contained additional compounds that might interfere with metabolic responses, thereby compromising the biological safety of the study. For this reason, a non-supplemented control group was used. However, this design choice may have limited the level of blinding. Future studies should consider developing more suitable placebo alternatives to ensure full blinding and further strengthen the internal validity of similar interventions.

## Figures and Tables

**Table 1:** Changes in lipid profile parameters in the groups between the pre-test and post-test.

Parameter (Unit)	Group	Pre-Test Mean ± SD	Post-Test Mean ± SD	Group	Time	Group × Time	Experiment Time Effect	
							$\eta_{p}^{2}$	Δ (%)
LDL Cholesterol (mg/dL)	Control	118.12 ± 14.37 [112.86–123.14]	115.84 ± 12.91 [111.22–120.46]	0.051	0.022	0.078	0.045	−8.03
	Experiment	120.04 ± 15.26 [114.54–125.46]	110.39 ± 13.11 ^†^ [105.31–114.49]					
HDL Cholesterol (mg/dL)	Control	46.14 ± 5.36 [44.08–47.92]	48.21 ± 5.92 [45.88–50.12]	0.162	0.014	0.231	0.055	+10.87
	Experiment	45.23 ± 5.47 [43.04–46.96]	50.15 ± 5.09 ^†^ [48.18–51.82]					
Triglycerides (mg/dL)	Control	178.39 ± 33.29 [166.09–189.91]	174.18 ± 31.22 [162.83–185.17]	0.131	0.046	0.072	0.043	−10.83
	Experiment	180.31 ± 32.12 [168.86–191.14]	160.77 ± 27.98 ^†^ [150.76–170.78]					

**Note:** All values are presented as means and standard deviations. LDL, low-density lipoprotein; HDL, high-density lipoprotein; Δ (%), percentage change; ^†^, significantly different from the pre-test values.

**Table 2:** Changes in inflammatory marker and antioxidant levels in the groups between the pre-test and post-test.

Parameter (Unit)	Group	Pre-Test Mean ± SD	Post-Test Mean ± SD	Group	Time	Group × Time	Experiment Time Effect	
							$\eta_{p}^{2}$	Δ (%)
CRP (mg/L)	Control	5.62 ± 1.38 [4.51–5.49]	5.48 ± 1.21 [4.57–5.43]	0.055	0.002	0.122	0.120	−41.13
	Experiment	5.81 ± 1.33 [4.52–5.48]	3.42 ± 0.91 ^†^ [2.67–3.33]					
IL-6 (pg/mL)	Control	10.35 ± 2.12 [9.24–10.76]	9.92 ± 2.21 [8.21–9.79]	0.255	0.016	0.082	0.085	−30.81
	Experiment	10.45 ± 2.24 [9.20–10.80]	7.23 ± 1.51 ^†^ [6.46–7.54]					
TNF-α (pg/mL)	Control	18.55 ± 3.21 [16.85–19.15]	18.22 ± 3.11 [16.89–19.11]	0.421	0.019	0.066	0.090	−27.06
	Experiment	18.40 ± 3.15 [16.87–19.13]	13.42 ± 2.64 ^†^ [12.06–13.94]					
Glutathione (µmol/L)	Control	10.28 ± 2.44 [9.13–10.87]	10.35 ± 2.51 [9.10–10.90]	0.311	0.031	0.222	0.130	+15.11
	Experiment	10.52 ± 2.22 [9.21–10.79]	12.11 ± 2.01 ^†^ [11.28–12.72]					
Malondialdehyde (nmol/L)	Control	4.63 ± 1.12 [4.23–5.03]	4.51 ± 1.03 [4.14–4.88]	0.210	0.005	0.091	0.135	−33.18
	Experiment	4.52 ± 1.01 [4.16–4.88]	3.02 ± 0.81 ^†^ [2.71–3.29]					

**Note:** All values are presented as means and standard deviations. CRP, *C*-reactive protein; IL-6, interleukin-6; TNF-α, tumor necrosis factor alpha; Δ (%), percentage change; ^†^, significantly different from the pre-test values.

**Table 3:** Changes in neurotransmitter and biomarker levels in the groups between the pre-test and post-test.

Parameter (Unit)	Group	Pre-Test Mean ± SD	Post-Test Mean ± SD	Group	Time	Group × Time	Experiment Time Effect	
							$\eta_{p}^{2}$	Δ (%)
BDNF (ng/mL)	Control	12.33 ± 2.54 [11.09–12.91]	12.16 ± 2.33 [11.17–12.83]	0.275	0.011	0.350	0.099	+12.12
	Experiment	13.53 ± 2.14 [12.23–13.77]	15.17 ± 2.07 ^†^ [14.23–15.74]					
Homocysteine (µmol/L)	Control	12.86 ± 2.32 [11.17–12.83]	12.74 ± 2.12 [11.24–12.76]	0.495	0.026	0.162	0.045	−10.53
	Experiment	12.53 ± 2.42 [11.13–12.87]	11.21 ± 2.14 ^†^ [10.23–11.77]					
Dopamine (ng/dL)	Control	220.06 ± 40.13 [205.69–234.31]	225.14 ± 42.17 [209.97–240.03]	0.281	0.046	0.313	0.133	+18.98
	Experiment	210.21 ± 38.24 [196.40–223.60]	250.11 ± 45.09 ^†^ [233.90–266.10]					
Serotonin (ng/mL)	Control	125.17 ± 25.03 [116.05–133.95]	128.11 ± 27.04 [118.34–137.66]	0.198	0.031	0.159	0.081	+16.56
	Experiment	120.13 ± 22.06 [112.13–127.87]	140.03 ± 28.09 ^†^ [129.98–150.02]					

**Note:** All values are presented as means and standard deviations. BDNF, brain-derived neurotrophic factor; Δ (%), percentage change; ^†^, significantly different from the pre-test values.

**Table 4:** Changes in physical performance parameters in the groups between the pre-test and post-test.

Parameter (Unit)	Group	Pre-Test Mean ± SD	Post-Test Mean ± SD	Group	Time	Group × Time	Experiment Time Effect	
							$\eta_{p}^{2}$	Δ (%)
BP 1RM (kg)	Control	80.4 ± 12.5 [75.71–84.29]	82.1 ± 11.7 [78.06–85.94]	0.061	0.003	0.099	0.112	+13.57
	Experiment	78.8 ± 13.2 [73.28–82.72]	89.5 ± 12.4 ^†^ [84.56–93.44]					
Squat 1RM (kg)	Control	120.7 ± 15.4 [114.49–125.51]	123.2 ± 14.6 [117.78–128.22]	0.075	0.002	0.058	0.125	+9.72
	Experiment	119.3 ± 14.8 [113.70–124.30]	130.9 ± 13.5 ^†^ [125.17–134.83]					
LS (kg)	Control	200.6 ± 22.3 [192.02–207.98]	205.2 ± 20.9 [197.52–212.48]	0.058	0.004	0.063	0.108	+15.21
	Experiment	195.8 ± 21.7 [187.23–202.77]	225.6 ± 19.5 ^†^ [218.02–231.98]					
HGS (kg)	Control	45.2 ± 7.4 [42.35–47.65]	46.1 ± 6.8 [43.57–48.43]	0.056	0.007	0.125	0.097	+10.51
	Experiment	44.7 ± 6.9 [41.53–46.47]	49.4 ± 6.6 ^†^ [46.64–51.36]					
CMJ (cm)	Control	35.8 ± 5.3 [33.10–36.90]	36.9 ± 4.7 [34.32–37.68]	0.080	0.002	0.199	0.123	+11.46
	Experiment	34.9 ± 6.1 [31.82–36.18]	38.9 ± 5.5 ^†^ 36.03–39.97]					
Sprint 10 m (s)	Control	1.80 ± 0.15 [1.62–2.08]	1.78 ± 0.14 [1.64–2.05]	0.059	0.006	0.358	0.110	−7.14
	Experiment	1.82 ± 0.14 [7.75–2.01]	1.69 ± 0.13 ^†^ [1.64–1.80]					
RSI (m/s)	Control	1.52 ± 0.22 [1.33–1.59]	1.63 ± 0.21 [1.49–1.73]	0.082	0.005	0.210	0.115	+14.96
	Experiment	1.47 ± 0.23 [1.41–1.59]	1.69 ± 0.20 ^†^ [1.58–1.76]					
IAT (s)	Control	17.54 ± 1.23 [16.56–17.44]	17.32 ± 1.15 [16.56–17.41]	0.095	0.003	0.411	0.120	−9.63
	Experiment	17.89 ± 1.18 [16.58–17.42]	17.07 ± 1.06 ^†^ [16.62–17.38]					

**Note:** All values are presented as means and standard deviations. 1RM, one repetition maximum; BP, bench press; LS, leg strength; HGS, hand grip strength; CMJ, counter movement jump; RSI, reactive strength index; IAT, Illinois agility test; Δ (%), percentage change; ^†^, significantly different from the pre-test values.

## Data Availability

The datasets generated and analyzed during the current study are not publicly available due to participant privacy concerns, but are available from the corresponding author upon reasonable request.
